# Chest Wall Pain after Minor Trauma

**DOI:** 10.5811/cpcem.2020.6.46961

**Published:** 2020-07-20

**Authors:** Deepak Chandwani, Jeff Arnold, John Terrusa

**Affiliations:** * California Task Force 6 - Urban Search and Rescue, Riverside, California; † Arrowhead Regional Medical Center, Department of Emergency Medicine, Colton, California; ‡ California Task Force 1 - Urban Search and Rescue, Los Angeles City Fire Department, Los Angeles, California

**Keywords:** Mondor’s disease, superficial thrombophlebitis, chest pain

## Abstract

**Case Presentation:**

A 30-year-old healthy male presented with a complaint of chest pain after mild thoracic trauma sustained while rescuing stranded flood victims during Hurricane Harvey. Careful physical examination revealed a tender palpable cord along the lateral aspect of his chest consistent with a superficial thrombophlebitis.

**Discussion:**

Mondor’s disease is a superficial thrombophlebitis with myriad underlying causes that can involve the thoracic wall. Although Mondor’s disease has been well described in the literature, this case describes a unique presentation in an austere environment with blunt trauma as the underlying cause.

## CASE PRESENTATION

While deployed during Hurricane Harvey with a Federal Emergency Management Agency Task Force, a 30-year-old male presented to the medical team for left-sided chest pain. He had been leaning over the rail of a military truck during search and rescue operations and developed pain and a “pulling sensation” when moving his left upper extremity. He had no significant past medical history and was well appearing with normal vital signs. Examination of the chest revealed a tender palpable cord along the left anterolateral chest wall without overlying erythema or warmth (Image).

## DISCUSSION

This case highlights a presentation of Mondor’s disease secondary to blunt trauma in a unique, austere environment. Mondor’s disease is a superficial thrombophlebitis first described by Charles Fagge in 1870 and later described by French surgeon Henri Mondor in 1939.^1^,^2^,^3^ Initially, the diagnosis referred specifically to superficial thrombophlebitis of the lateral thoracic, thoracoepigastric, or superior epigastric veins of the thoracoabominal wall. Currently, the diagnosis has expanded to include thrombosis of the dorsal penile vein.^2^,^4^

The underlying etiology of Mondor’s disease is varied and in many cases unknown. It can be related to trauma, physical activity, breast surgery, and rarely breast carcinoma.^5^ It is thought that an initial injury to the vein leads to inflammation, thrombosis, and fibrosis, although some cases are believed to be related to lymphangitis.^1^,^2^ Symptoms typically last 4–8 weeks with spontaneous resolution. Treatment consists of non-steroidal anti-inflammatory drugs and warm compresses. It is imperative that possible underlying causes are considered such as disease processes that result in a hypercoagulable state, vasculitis/vascular diseases, carcinoma and, in the case of penile Mondor’s disease, sexually transmitted diseases.^2^ Diagnosis is generally based on clinical presentation; however, it can be confirmed with ultrasound.

**CPC-EM Capsule**

What do we already know about this clinical entity?

Mondor’s disease is a superficial thrombophlebitis that can involve the chest wall, causing pain and discomfort.

What is the major impact of the image(s)?

This case highlights the importance of careful physical examination in a patient with chest pain.

How might this improve emergency medicine practice?

Increased awareness of Mondor’s disease may lead to accurate diagnosis and appropriate therapeutic intervention, potentially minimizing unnecessary testing.

## Figures and Tables

**Image:**
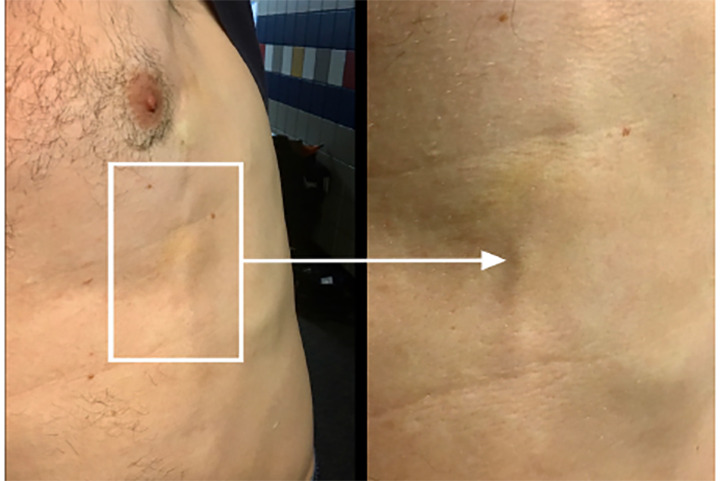
Subcutaneous cord along the anterolateral thoracoabdominal wall.
